# Motor patterns during active electrosensory acquisition

**DOI:** 10.3389/fnbeh.2014.00186

**Published:** 2014-05-28

**Authors:** Volker Hofmann, Bart R. H. Geurten, Juan I. Sanguinetti-Scheck, Leonel Gómez-Sena, Jacob Engelmann

**Affiliations:** ^1^Active Sensing, Faculty of Biology, Cognitive Interaction Technology – Center of Excellence, Bielefeld UniversityBielefeld, Germany; ^2^Cellular Neurobiology, Schwann-Schleiden Research Centre, Georg-August-UniversitätGöttingen, Germany; ^3^Sección Biomatemática, Laboratorio de Neurociencias, Facultad de Ciencias, Universidad de la RepublicaMontevideo, Uruguay; ^4^Bernstein Center for Computational Neuroscience, Humboldt Universität BerlinBerlin, Germany

**Keywords:** electric fish, electrolocation, motor patterns, sensorimotor interaction, behavior, quantitative behavioral analysis, sensory flow

## Abstract

Motor patterns displayed during active electrosensory acquisition of information seem to be an essential part of a sensory strategy by which weakly electric fish actively generate and shape sensory flow. These active sensing strategies are expected to adaptively optimize ongoing behavior with respect to either motor efficiency or sensory information gained. The tight link between the motor domain and sensory perception in active electrolocation make weakly electric fish like *Gnathonemus petersii* an ideal system for studying sensory-motor interactions in the form of active sensing strategies. Analyzing the movements and electric signals of solitary fish during unrestrained exploration of objects in the dark, we here present the first formal quantification of motor patterns used by fish during electrolocation. Based on a cluster analysis of the kinematic values we categorized the basic units of motion. These were then analyzed for their associative grouping to identify and extract short coherent chains of behavior. This enabled the description of sensory behavior on different levels of complexity: from single movements, over short behaviors to more complex behavioral sequences during which the kinematics alter between different behaviors. We present detailed data for three classified patterns and provide evidence that these can be considered as motor components of active sensing strategies. In accordance with the idea of active sensing strategies, we found categorical motor patterns to be modified by the sensory context. In addition these motor patterns were linked with changes in the temporal sampling in form of differing electric organ discharge frequencies and differing spatial distributions. The ability to detect such strategies quantitatively will allow future research to investigate the impact of such behaviors on sensing.

## Introduction

The term active sensing refers to the use of energy for sensing with the energy being emitted from the animal itself. Echolocation in bats and marine mammals, active electrolocation in fish, whisking in rodents and haptic touch are among the classic examples of active sensing (Nelson and MacIver, 2006; Grant et al., 2014). In a more general framework sensing is increasingly being considered as inevitably active, with motor activity and sensing being intertwined. Thus, active sensing relies on the coordination of motor control and sensory processing, e.g., by structuring motor activity in more or less stereotyped sensing movements, termed active sensing strategies (Schroeder et al., 2010; Hofmann et al., 2013b).

Here we focus on motor strategies in a model organism for active sensing, the weakly electric fish *Gnathonemus petersii* (Mormyridae). This fish generates a three dimensional dipole field around its body by emitting short currents pulses (electric organ discharges = EOD) from its electric organ (Lissmann, 1951, 1957; Machin and Lissmann, 1958; Harder et al., 1964). While the waveform and amplitude of these EODs are fixed, their inter-pulse-interval can be varied voluntarily by the animal. The modulation of the EOD frequency (fEOD) can be experimentally exploited to investigate perceptual properties in the electrosensory system (Toerring and Moller, 1984; Post and von der Emde, 1999; Schwarz and von der Emde, 2001; Caputi et al., 2003). Each EOD, building a three dimensional electric dipole field, results in a distribution of currents across the skin of the animal, where electroreceptors for sensing are situated (Castelló et al., 2000; Bacelo et al., 2008). The electric field is distorted by objects close to the animal that differ in electrical properties from the surrounding water. As a result the pattern of current distribution at the skin is changed, which is termed *electric image* (EI) (Rother, 2003; Rother et al., 2003). This modulation, is the crucial sensory input in electrolocation and the animals rely on the processing of EIs for electro location and navigation (Graff et al., 2004; Von der Emde et al., 2010; Von der Emde and Engelmann, 2011).

Several features make weakly electric fish especially fascinating when one tries to understand how sensing and moving can be joined in an effort to explore the world. In contrast to other sensory systems where this interaction has been explored in depth, such as vision in insects (Hyslop et al., 2010; Fotowat and Gabbiani, 2011; Longden and Krapp, 2011; Egelhaaf et al., 2012), active electroreception has been shown to be omnidirectional (Snyder et al., 2007). While the photoreceptors of insect eyes are densely clustered, electroreceptors are distributed across a vast portion of the animal's body and receive stimuli from all directions. This lack of directionality in the electroreceptors makes an electroreceptor sensitive to modulations of the local field amplitude originating from any direction. In addition to these aspects of omnidirecitionality, peripheral specializations of the electroreceptor distribution equivalent to multiple electrosensory fovea have been found in this sensory system (Castelló et al., 2000; Bacelo et al., 2008; Hollmann et al., 2008; Pusch et al., 2008). Omnidirectional sensing and differentiation of the sensory mosaic may have led to the emergence of motor strategies that aid in the extraction of sensory features over the entire ensemble of sensors on the one hand- or sub- populations of sensors on the other (von der Emde and Schwarz, 2002). Active sensing strategies have indeed been shown to be potentially beneficial in specific tasks (Solberg, 2009; MacIver et al., 2010; Silverman et al., 2013). A second aspect that makes weakly electric fish an interesting model organism to study sensorimotor interaction is that the space over which they can sense is well matched by the space through which they can move (Snyder et al., 2007). Again this shows the tight coupling between the sensory and motor domains. Finally the pulsatile nature of the Mormyrid electrolocation system offers the experimental opportunity to precisely determine the point in time when sensory input is acquired. This not only offers a measure of an animals' attention (Hall et al., 1995; Post and von der Emde, 1999; Caputi et al., 2003), but also greatly reduces the uncertainty in analyzing the sensory flow generated during electrolocation. By integrating the temporal EOD pattern with the body kinematics, the resulting electrosensory flow can be modeled for natural behaviors at high precision: each EOD generates a “sensory snapshot” that can be expected to be of behavioral relevance. In our experimental paradigm fish were socially isolated. Therefore, communication most likely plays a subordinate role and we expect the majority of EODs being emitted in the context of electrolocation. Hence, the same is true for the sensory input based on each of these snapshots.

What a fish perceives depends on the nearby environment as well as the animal's position, posture and EOD history. Moreover, the movement of the animal between successive EODs can account for the generation of cues in the resulting spatiotemporal change of the electrosensory input over time, i.e., electric flow (Sim and Kim, 2011; Fotowat et al., 2012; Hofmann et al., 2013a). By relating behavioral kinematics with the ensuing electrosensory flow, we aim to identify active sensing strategies that facilitate the processing of sensory information in this omnidirectional near-range sensory system. To achieve this, the highly complex motor patterns need to be quantified to allow a reduction of data complexity and a formalized pattern analysis. We here describe prototypical movement components by performing a cluster analysis (Braun et al., 2010; Geurten et al., 2010) based on kinematic data (thrust, slip and yaw velocities) of individual *G. petersii* exploring resistive objects under unrestrained conditions. Following this segmentation, we detected and analyzed reoccurring stable combinations of kinematic classes to describe behavioral sequences. We will use the term motor patterns when we describe the results of these analyses in the following while we use the term electromotor patterns when we report data on EODs.

Active sensing behaviors in electrolocation have been described in pioneering studies (Toerring and Belbenoit, 1979; Toerring and Moller, 1984; Von der Emde, 1992). However, these were based on semi-quantitative human observer-based classifications, which required a priori assumptions about the relevance and structure of behavioral patterns. We here show that similar results can be obtained using a quantitative approach based on objective and therefor reproducible criteria. In future, the quantitative detection of specific behaviors, which is comparable to a quantitative form of an ethogram (Cavraro et al., 2013), should be combined with the modeling of the sensory input, which will allow to quantify the sensory input during active electrolocation in depth and to extract principles of active sculpting of sensory flow in omnidirectional sensory systems. Moreover this characterization of the natural sensory input dynamics will offer the chance to study the sensory processing on the neuronal level using dynamically changing inputs of behavioral relevance both in neurophysiological and in modeling approaches. Combining this kinematic data with modeling the electrosensory input will enable us to characterize the role of “active sensing strategies” in shaping the electrosensory flow.

## Materials and methods

In order to analyze sensory relevant behavior we recorded *Gnathonemus petersii* (*N* = 11, length 11 ± 1 cm) during unrestrained object exploration. The videos were stored on a computer and analyzed offline. All data recordings and offline analysis procedures were carried out using custom written MATLAB routines (v. R2011b). If not noted otherwise, data are reported as medians with the sample size denoted by n and the number of individual fish by *N*.

### Video recordings

Fish were recorded in a Perspex® arena (80 × 80 × 15 cm). The arena was set up in a visually and acoustically isolated, separate room and filled with 6 ± 0.5 cm of water (water conductivity: 100 μs ± 10; temperature: 23 ± 2°C). Two plastic tubes (*r* = 3 cm; l = 12 cm) were placed alongside two opposing walls of the tank and served as shelters. Eight silver wire electrodes, connected to a differential amplifier (custom build), were placed in the corners and in the middle of each wall, approximately 1 cm above the tank's floor to record the animals EOD signals (see Figure S1 for a sketch of the setup). A camera (AVT Marlin F-033B, Stemmer Imaging, 656 × 494 pixels, 12 bit, maximal frame rate 78 Hz) was set up approx. 80 cm above the tank to record images of the central area of the tank (64 × 48 cm). Frame grabbing was synchronized to the animals EODs and video recording were started automatically if an animal moved into the central arena. All videos were obtained after sundown with the only light source being a grid of IR-LEDs (880 nm) below the arena. *Gnathonemus* was shown to be incapable of navigating visually under these lighting conditions (Ciali et al., 1997; Landsberger et al., 2008).

Before and after experiments fish were housed in a large tank that was divided to house individual fish. The partitions allowed the animals to experience conspecifics electrically (water conductivity: 100 μs ± 10, temperature 23 ± 2°C, 12/12 light/dark). After being transferred to the experimental tank, fish were allowed to adapt for 48 h. Following this acclimation phase video recordings were performed over the following five nights. Three differently sized metal cubes of 1, 8 or 27 cm^3^ (edge length 1, 2, and 3 cm) were used with the mid-sized cube presented in two orientations (0 and 45°). The four object conditions and one night without an object being present were recorded in a random order. The objects (if present) were placed into the middle of the tank directly after sunset and were removed in the morning after each recording.

### Offline analysis

For offline analysis we used the first 50 videos of each night in which a fish entered the center of the tank in order to prevent strong effects of spatial memory and habituation. We tracked the animals' position, its orientation (position of head vs. tail), and posture based on contrast methods according to the following steps of a custom written automatized tracking procedure: At first all frames in which the fish was not or not completely in camera view were removed. After that, we subtracted the background of the image and used a threshold operation to transfer it to a black and white image. To detect the fish position a centroid was calculated for the extracted shape of the fish. To obtain the body's curvature we fitted a 3rd order polynomial to the midline of the extracted shape of the animal. The fit was constrained by the previously determined length of each individual animal. The head and tail were discriminated by comparing the mean intensity of a constant area centered on both ends of the animals' black and white silhouette. Since the head, compared to the tip of the tail, in general was more bulky, the area with the higher level of black was determined to be the head.

**Figure 1:**
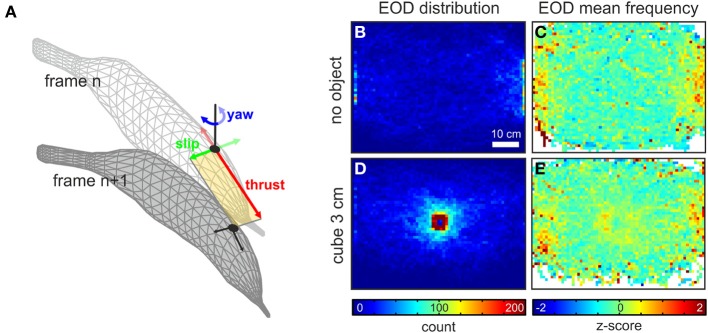
**Data extraction from behavioral recordings and spatial aspects of EOD behavior. (A)** Principle of the extraction of the kinematic data between two successive video frames. The fish's head position (black dots) was extracted for each frame. Based on the body orientation, determined from a fit through the animal's midline, the current heading (orientation) was determined. From this frame of reference (yellow area) the amplitude of the thrust and slip (red and green arrows) was determined as the vectorial components between the current and the successive frame (frame *n* and frame *n* + 1). The yaw component (blue arrows) was calculated as the difference in angle between the headings of successive frames. **(B**,**C)** Spatial occurrence **(B)** and mean fEOD **(C)** for data obtained in absence of an object (*n* = 46230 frames, 11 fish). The data is plotted as two-dimensional histograms using a bin width of 1 cm^2^. The spatial occurrence is shown as the cumulative count of frames in each bin. The fEOD is calculated as the mean frequency of all frames in a bin. Frequency data was z-transformed in order to allow the comparison across different nights. **(D**,**E)** Same as **(B,C)** for data with a metal cube of 3 cm side-length presented in the center of the arena (*n* = 69759, 11 fish).

Next we determined the available 2-D body kinematics (thrust, slip and yaw velocities). The body orientation was obtained frame-wise by using the anterior 25% of the body as a frame of reference. Thrust and slip components were determined as the difference of head position between successive frames along the axes of this frame of reference (see Figure 1A). The yaw component of the movement was determined as the difference in angle between the orientations in two consecutive video frames. The velocities were calculated based on the distances traveled between two successive video frames. As the individual frames of the videos were triggered by the emission of the fish's EODs, the time base for this calculation was variable. On average EODs were emitted at 25 Hz. At the average swim speed of about 10 cm s^−1^ we thus were able to resolve at approximately 0.4 cm s^−1^ resolution. In the context of our aim to characterize general kinematic patterns, this resolution was considered to be sufficient. If one were interested in a more detailed description of the kinematics, higher sampling rates would be more suitable. As we here as mainly interested in the direct coupling of electromotor and motor patterns, we decided to sample the kinematics in the natural time-frame, i.e., at the fishes own sampling interval.

**Figure 2:**
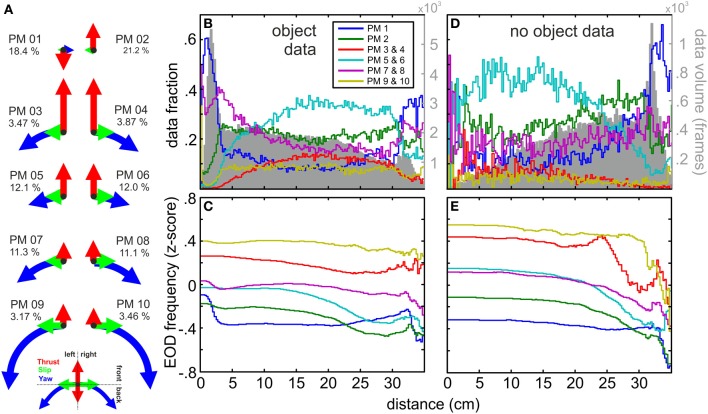
**Kinematic composition of prototypical movements (PMs), spatial distribution and relation to fEOD. (A)** Normalized thrust, slip and yaw velocity amplitudes for centroid values of the 10 clusters and the data fraction of each PM relative to the size of the entire dataset (*n* = 277585). PMs 3–10 occurred in mirror-imaged pairs with respect to their slip and yaw velocity directions. PM 1 and PM 2 were mainly thrust dominated; note that thrust was negative for PM 01. Mirror-imaged PMs occurred equally likely (see percentage of each PM). PMs 3–10 describe a gradual transition from PMs with maximal translational amplitude (PM 03/04) toward those of maximal rotational amplitude and reduced thrust (PM 09/10). **(B)** Relative spatial distribution of the PMs plotted against the Euclidian distance to the object for nights in which an object was placed in the tank (*n* = 231355 frames). PM 01 and PM 07/08 were the most common PMs close to the object, while the other PMs declined in their relative frequency. The absolute volume of data per bin is indicated by the gray histogram in the background. **(C)** Mean fEOD (z-scored) associated with the mirror-imaged PM pairs and PMs 01 and 02 as a function of the Euclidian distance to the object. The data fraction used at each distance can be seen in the gray histogram in **(B)**. **(D,E)** Same as **(B,C)** but for nights without an object present (*n* = 46230). Note that the spatial distributions of PMs and the absolute data per bin were inverted compared to the data obtained in presence of an object. The EOD frequencies associated with PMs were fairly comparable for both conditions (compare **E,D**). Note that on average the PMs had significantly (see text) different EOD frequencies. In general PMs with higher kinematic amplitudes (PM 03/04 and PM 09/10) occurred with higher EOD frequencies. The slower translational PMs 01 and PM 02 had significantly lower EOD frequencies. While most PMs had a stable fEOD with object distance, fEOD increased with nearness to the object for PM 01 and less so for PMs 07 and 08.

Based on the body kinematics we performed a three dimensional cluster analysis, previously established and used for the investigation of the flight structure in different insect species (Braun et al., 2010, 2012; Geurten et al., 2010). In the following we describe briefly how we treated our data under consideration of the ideas described in the preliminary studies. To normalize for the different dimensionality of rotational and translational kinematics, the data was z-scored. Here, thrust and slip were subjected to the z-scoring together. Next, a hierarchical clustering (squared Euclidian distance | wards criterion) was performed in order to determine the possible number of clusters. For the determined range we used the MATLAB implementation of the k-means cluster algorithm with a squared Euclidian distance and preclustering instead of random start conditions (Arthur and Vassilvitskii, 2007) to cluster the data. The results of cluster runs for *k* = 2 to *k* = 50 were *post-hoc* evaluated for their stability and quality (Figure S2) as published by Braun et al. (2010) rendering a number of 10 centroids. From there on the values of the centroids were used to express the kinematic properties of the different clusters, which are termed “prototypical movements” in the following “PMs” (see Figure 2A) and resemble the basic movements that were executed between frames. By this categorization the complexity of the dataset was reduced to building blocks of the animal's kinematics.

In the context of understanding behavior, the prototypes themselves differentiate the activity of an animal on a comparatively short timescale. Thus, in order to be able to detect and discriminate different types of behavior we extended the analysis beyond kinematic frames and investigated relationships of consecutive PMs. Here we started with the simplest possibility, which is to analyze the occurrence, length and duration of homogenous chains of single PMs (e.g., chain of frames in which an animal remained in a specific PM for some defined time). As a second approach we calculated the transition probabilities between PMs by comparing the frequency of occurrence of a transition from one PM to another with the overall frequency of occurrence of the target PM. For these calculations the complexity of the data was reduced to transitions only. The resulting probabilities were used in a hidden Markov Model to calculate the most frequently occurring sequences of PM transitions, termed “super-prototypical movements” (SPMs). This is based on the probability matrix obtained from the Markov Model. This matrix describes the transition probability from any PM to any other given PM. From this we calculated the overall probability for every chain consisting of five PM transitions—a so called super-prototypical-movement (SPM). By ordering all SPMs of a given chain length by their overall probability we then determined the 100 most probable SPMs. After this we further reduced the complexity of these transition sequences by removing the directional information contained in the slip and yaw velocity. To do so, we merged pairs of PMs of otherwise mirror-image like kinematics. Furthermore we combined sequences that were constructed from the same PMs (in number) but differed slightly in their fine structure (e.g., A-A-A-B-A-A and A-A-B-A-A-A) into single SPM. These steps led to a reduced set of 17 frequently reoccurring SPMs (Figure S3). These were analyzed with respect to their occurrence in object and non-object data. Finally, for those SPMs of interest, we used spatial conditionals (e.g., nearness to an object), to extract specific subsets of sequences having a specific spatial relation (see also results).

The fEOD was z-scored across recording nights in order to account for changes in basal emission frequency of individual fish between days.

## Results

We here report data from the first 50 videos of eleven fish (*N* = 11) recorded in 55 experimental nights with a metal object being present in the experimental arena in 44 nights. After excluding frames in which fish were not completely in view (see Materials and Methods) the data set explored consists of more than 2500 video sequences (*n* = 277585 frames, ≈ 205 min). Data did not differ between the different object sizes and object orientations. Therefore, if not mentioned otherwise, our presentations include data from all of those. We will first detail the results of a kinematic prototyping, and then show that these prototypes can be used with varying levels of complexity to determine and extract consistent behavioral sequences from the data. The results will be presented at different levels of complexity. On the single-frame level we quantified the kinematic components of body movements which were classified into different categories of “prototypical movements” (see Methods). This classification is independent of the temporal sequence of the data. When using these prototypes to extract longer kinematic chains of consecutive frames, the temporal sequence was considered. Such sequences are referred to as a “behavior” throughout the manuscript. Using a structured behavioral sequence, we show that electrosensory object scanning behavior is mainly characterized by a succession of several of these behaviors.

In absence of an object, EODs were most frequently emitted in positions close to the shelters with a nearly uniform distribution in the remaining parts of the arena (Figure 1B and gray histogram in Figure 2D). In presence of the metal cubes this changed dramatically to an object centered distribution (Figure 1D and gray histogram in Figure 2B). The mean fEOD, indicative of the fishes arousal or attentional state (Toerring and Moller, 1984; Caputi et al., 2003; Carlson and Hopkins, 2004), showed an almost equal level throughout the arena with fEOD increases nearby the shelters (Figures 1C,E). In presence of an object the mean EOD frequencies were increased close to the object in the center of the arena (Figure 1E). Although this shift of the fEOD was restricted to the region surrounding an object, it lead to a slight shift of the global median EOD-frequencies (median fEOD with object: 26.3 Hz, *n* = 231355; median fEOD without object: 26.1 Hz, *n* = 46230; *p* = 0.01, Mann-Whitney *U*-test).

### Prototypical movements

To quantitatively describe different behaviors on a frame-by-frame level we clustered the kinematic data (Braun et al., 2010; Geurten et al., 2010). Based on quality and stability considerations, we obtained ten clusters with their corresponding centroids being referred to as “prototypical movements” (“PMs”; Figure 2A). These PMs aggregate single-frame kinematic data into clusters and serve to determine reappearing kinematic components. We found two PMs to be dominated by translational velocities (PM 03 and PM 04) and two in which rotational components dominated (PM 09 and PM 10). PMs 05–08 constituted transitions between these extremes. PMs 03–10 occurred in pairs with comparable thrust velocities, but opposing directions in the slip- and yaw-velocity. This was different for PM 01 and PM 02 which were dominated by the thrust-velocity with only a small contribution of slip- and yaw-velocity. PM 01 was the only prototypical movement with a negative average thrust-velocity, whereas the thrust-velocity of PM 02 was comparable to PMs 05–08, characteristic for movements with intermediate velocities. In the following the symmetric pairs of PMs are pooled if not noted otherwise. PMs describing extreme movements, e.g., PMs 09/10 with extreme yaw or PM 03/04 with extreme thrust occurred with lowest probability. In contrast to this, PMs with moderate or intermediate movements had a comparatively high data fraction. Mirror-symmetric PMs in all cases had comparable data fraction.

It is important to note that the illustration of the PMs in Figure 2 are based on the centroid values of the clustered data and that within each cluster considerable spread around these centroids occurred (see also Figure S3). Nonetheless, PMs were clearly different in their spatial distribution (Figures 2B,D): For nights in which an object was presented in the tank PMs with low thrust velocities (PM 01 and PM 07/08) occurred more frequent close to the object to the shelters while the other PMs occurred less frequently (Figure 2B). In absence of an object PM 01 was most frequently observed close to the shelters while the PMs dominated by positive thrust increased toward the center of the arena (Figure 2D). This demonstrates that the animals' swim pattern was altered close to an object (<5 cm) showing reduced swim speed and increased stationary or backwards kinetics the close the fish was to the object. These differences were associated with differences in electromotor parameters, i.e., the EOD frequencies between data from different PMs differed significantly (*p* < 0.05, Kruskal-Wallis test, H-corrected = 7363.2, *F* = 972.9). While EOD frequencies differed between the groups (median values: PM 03/04 35.4 Hz, PM 09/10 32.4 Hz vs. PM 01 23.2 Hz and PM 02 22.8 Hz, Dunn *post hoc* analysis, *p* < 0.05) it was indistinguishable within the pairs of mirror-imaged PMs. PMs containing higher velocities in their kinematic components on average were associated with higher EOD frequencies. For PMs 03/04, PM 05/06, and PM 09/10 the EOD frequencies were relatively stable with respect to distance to the object (Figures 2C,D), while PMs 01, 02, and 07/08 showed an slight increase of fEOD at small distances (Figure 2C). The finding that PMs differed between PMs was also found for nights without the object being present. The spatial distributions were comparable to those observed in presence of an object, although clear differences close to the shelter were found (compare Figures 2C,E). These differences are not further explored here, but are likely due to different behavioral states. In absence of an object fish mainly stayed close to the shelter and were less active, and EODs of lower and less variable frequencies are characteristic for resting Mormyrids (Moller et al., 1979).

**Figure 3:**
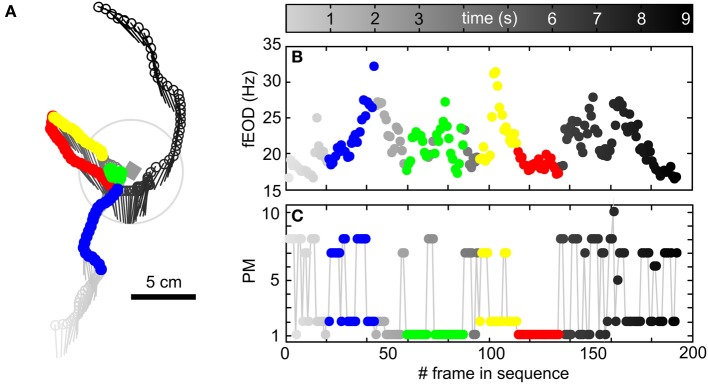
**Exemplary trajectory of an animal including quantitatively characterized behaviors, fEOD and the temporal structure of single-frames based PMs. (A)** Head position (dots) and orientation (lines) of an animal during a segment (192 frames, 9.16 s) of a video where a metal cube of 1 cm side length (gray rectangle) was present in the arena. The temporal structure of the behavioral sequence is represented by the gray level of the symbols (see color bar above **B**). The animal entered the view of the camera from south, approached the cube, stayed close to the cube and departed with a north-westerly heading. This was followed by a return to the object through backwards swimming, a bent around the object and then finally the animal left the arena in a northward direction. The color coded segments of this trajectory show examples of the kinematic chains characterized quantitatively in the following (“object approach” in blue, “stationary behavior” in green, “backwards swimming” in red, “object departure” in yellow). **(B)** fEOD of the trajectory shown in **(A)**. Note that during the object approach the EOD train accelerated markedly, while it was variable during the “stationary behavior” and was low and regularized during “backwards swimming.” **(C)** Sequence of prototypical movements occurring throughout the trajectory shown in **(A)**. For details regarding the classification of the quantified behavioral sequences, please see main text.

**Figure 4:**
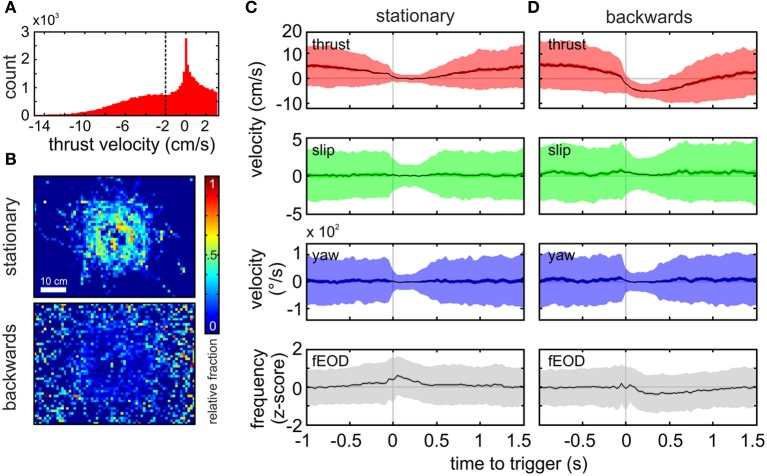
**Spatial and kinematic characterization of “stationary behavior” and “backwards swimming” patterns. (A)** Histogram of the thrust velocity of all frames classified as PM 01 (*n* = 50931, bandwidth = 0.2 cm s^−1^). We chose a threshold of −2 cm s^−1^ to further subdivide chains of PM 01 into “stationary behavior” and “backwards swimming” chains. Homogenous chains with a minimum of 10 consecutive frames were analyzed exclusively. **(B)** Normalized spatial distribution of the “stationary behavior” (top, *n* = 6111 frames) and “backwards swimming” (bottom, *n* = 5252) chains. Note that stationary chains occurred more frequently at the object, while backwards swimming chains were distributed without clear peaks. (Bin size = 1 cm^2^). **(C**,**D)** Averages of kinematic velocities and fEOD triggered by the onset of the target PM chain for “stationary behavior” (**C**, 672 chains, *n* = 6111) and “backwards swimming” (**D**, 710 chains, *n* = 5252). From top to bottom the panels show the mean thrust velocity, the mean slip velocity, the mean yaw velocity and the z-scored mean fEOD. The dark shaded area surrounding the mean indicates the ±95% confidence interval of the mean, while the lighter shaded area indicates one standard deviation. For the “stationary behavior” **(C)** the thrust velocity decreased within the time prior to the start of the sequence (gray vertical line at *t* = 0), and then the average was close to zero, rising again after roughly half a second. Slip and yaw velocities remained fairly constant with a reduction in standard deviation within the kinematic chains, while the fEOD showed a slight increase with a peak at the beginning of the target chain. For the “backwards swimming” behavior **(D)**, the thrust velocity decreased comparatively later and more rapidly prior to the onset of the sequence. The thrust velocity remained below zero for about ¾th of a second. The slip and yaw profiles were comparable to those during stationary probing, whereas the fEOD differed with transiently decreasing just after the trigger point.

The single-frame based prototypical movements described thus far make up the building-blocks of behavior. This can be seen in Figure 3, where we show a single trajectory extracted from one of our videos recorded with a 1 cm^3^ metal cube in the center of the arena. In this video sequence of less than 10 s the animal showed various types of behavior that extend beyond simple PMs in both complexity and duration. Characteristic behavioral patterns like object approach, short stationary object-probing phases followed by backwards swimming alongside the object and a radial passage of the object can all be found in this exemplary sequence (Figure 3A). Over the 192 video frames of the example sequence shown, a variety of different PMs occurred (Figure 3C). Hence the extraction of behavioral sequences with sensory relevance cannot be achieved based on the analysis of single frame based characteristics of the PMs alone. Thus, to be able to detect and describe behaviors beyond the single frame level, we analyzed homogeneous series of PMs. As detailed in Offline analysis these chains proofed to be characteristic for motor patterns related to the inspection of objects as for example “stationary behavior” and “backwards swimming” behaviors (Figure 4). Homogeneous chains were also characteristic of more general locomotor kinematics (Figures S4, S5). To analyze more complex patterns (Behavior with heterogeneous kinematics), we calculated the transition probabilities between PMs and used a hidden Markov model to detect frequently occurring behaviors constructed from heterogeneous PM chains, so termed “super-prototypical movements” (SPMs, Figure S3). Finally we subjected such SPMs to spatial conditionals in order to select a subset of specific behaviors. This enabled us to select SPMs we consider to be linked to the active sensory exploration of the objects, in our case sequences where animals approached objects from far in a stereotyped approach.

### Behavior with homogeneous kinematics

For the analysis of homogeneous chains of a single PM we here focus on PM 01. The thrust in this PM spans from negative to slightly positive values (Figure 4A). In order to differentiate between negative thrust values from close to zero velocity values and to account for the apparent bimodal distribution, we separated chains of PM 01 by their mean thrust velocity using a threshold −2 cm s^−1^. PM 01 chains composed of at least ten consecutive PM 01 frames with the average thrust-velocity below the threshold were termed “backwards swimming.” In contrast those chains composed of at least ten consecutive PM 01 frames with a mean thrust velocity higher than the threshold were termed “stationary behavior.” Remarkably, although these behaviors were defined by their kinematic parameters only, they showed a markedly different spatial distribution. While “stationary behavior” most likely occurred next to the object, “backwards swimming” had a broader distribution that spread over the whole tank with a slight plateau of increased occurrence for distances <10 cm (Figure 4B upper vs. lower panel) which indicated the validity of the additional separation.

For “stationary probing” as defined above we found 672 chains (mean length 16.7 ± 9.7 frames, mean duration 0.83 ± 0.86 s). We obtained the average kinematic and electromotor parameters associated with these chains by calculating an average that was triggered on the start of the PM-chain (Figure 4C). From this average it was noticeable that the stationary phase was preceded by a gradually decrease in thrust velocity prior to time zero. After that, the thrust velocity remained close to zero for about 500 ms. During this period the standard deviation of the averaged thrust-, slip- and yaw-velocities were markedly reduced. The possible following transition to other kinematics was indicated with the gradual increase in the standard deviation and the rise of the average thrust velocity toward positive values. The “stationary behavior” was tightly linked with a slight increase of the average fEOD.

For chains with mean thrust velocity values below the threshold we found 710 kinematic chains of “backwards swimming” (mean length 22.5 ± 18.7 frames, average duration 1.15 ± 0.94 s). The triggered averages for “backwards swimming” (Figure 4D) differed from those of the “stationary behavior.” Here the thrust decreased more steeply prior the start of the sequence and remained in the negative range for an average period of more than 750 ms. Similar to “stationary behavior,” the average thrust velocity then returned to positive values. Contrary to “stationary behavior,” a slight decrease of the z-scored fEOD was found during the period of reversed thrust velocity. The clearly distinct EOD characteristics and the spatially differing occurrence of the kinematically characterized chains, both illustrate the validity of the post-clustering separation, as this enabled us to extract two functionally different types of behavior.

### Behavior with heterogeneous kinematics

**Figure 5:**
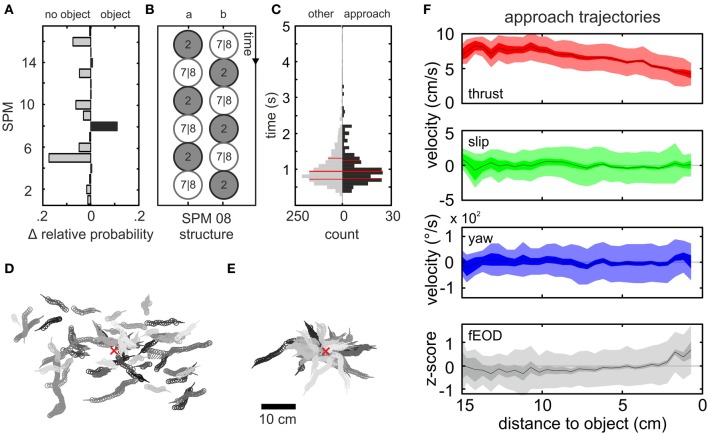
**Spatial and kinematic characterization of the “object approach” pattern. (A)** Difference in relative probability of occurrence of the 17 SPMs for data without (left) and with object (right) presented in the tank. Most SPMs occurred equally likely for both conditions, while some had a higher probability of occurrence in absence of an object. Only SPM 08 was found more frequently when an object was present and hence was further explored. **(B)** Schematic showing the different possible structures of SPM 08. Constant PM segments were reduced to a single frame represented by the circles in order to illustrate the inter-PM transitions. **(C)** Distribution of the duration of all SPM 08 chains. The data was split into SPMs in which the object was approached (black histogram, *n* = 4599 frames) and all remaining sequences of the SPM (gray histogram, *n* = 34436 frames). The distributions were indistinguishable and showed similar medians (thick red line, percentiles = thinner red lines). **(D**,**E)** Lollipop representation (dot = head position; handle = body orientation) of 50 randomly chosen SPM 08 trajectories **(D)**. **(E)** Fifty randomly chosen trajectories classified as SPM 08 following the application of spatial conditionals. These sequences are referred to as “object approach” behavior. In **(D,E)** individual behavioral chains are all plotted in the same consistent shade and the object center is indicated by the red x. Note that without the spatial filtering trajectories were randomly distributed, while the spatial conditional led to the extraction of object-approach sequences **(E)**. **(F)** Distance dependent averages (from top to bottom: thrust velocity, slip velocity, yaw velocity, z-score of fEOD, see also Figure 4, 235 sequences, *n* = 4599 frames) of the kinematic and fEOD parameters for the “object approach” chains of SPM 08. Deviating from the previous figure data here is displayed with respect to the distance between the animal and the center of the cube. A distance dependent decrease of the thrust velocity is noticeable starting at a distance of 10 cm. For close distances the fEOD transiently increased toward the object.

To construct robust and frequently occurring chains of non-homogeneous PMs, we used the transition probabilities between PMs in a “Hidden Markov Model” (Eddy, 1996; Krogh, 1998; Wallisch et al., 2009). We reduced the complexity of the 100 most frequent chains of 5 PM-transitions by excluding the directional information of the slip and yaw vectors and unifying chains of qualitative identical fine structure (see Materials and Methods). This procedure resulted in a set of 17 “super-prototypical movements” (SPMs, Figure S3). To determine if any of these SPMs was specifically related to the presence of an object, and thus might relate to sensory relevant kinematics, we calculated the relative frequency of occurrence for these 17 SPMs conditioned by the presence or absence of an object. The difference between both conditions is plotted in Figure 5A. SPM 08 exclusively showed a higher rate in the data obtained with an object, and thus was explored in more detail.

SPM 08 was characterized by repetitive transitions between PM 02 and PM 07/08 (Figure 5B), that formed a general mode of forward swimming. This behavior occurred with a broad spatial distribution across the tank showing highest probability at positions with a relative distance of about 5–8 cm to the cube (Figure 5D; coherent trajectories plotted in uniform shade). To select the subset of trajectories in which the cube was directly approached, we applied two spatial conditionals: the last frame of a SPM 08 chain needed to end within a distance of 4 cm (head-cube distance) and the distance to the cube needed to be smaller in this last frame than in the first frame of the sequence. This yielded 235 sequences (1937 SPM 08 chains without spatial filter) that we refer to as “object approach” chains (Figure 5E). Applying the same filter to the data without an object being, i.e., analyzing it as if an object would have been present, yielded zero chains. This indicates that by applying spatial filtering we were able to extract behavioral chains of clear object relation exclusively. The selected trajectories on average lasted 1.1 ± 0.51 s (mean ± *SD*), which was similar to the duration of SPM 08 chains without the spatial filtering applied (Figure 5C, gray vs. black bars). Compared to the other SPMs (Figure S3B), these were relatively long lasting sequences.

For the approaching behavior we again calculated the average kinematic and electromotor characteristics. To do so, we used the animals' relative distance to the cube as a frame of reference (Figure 5F). Slip- and yaw-velocities did change markedly, while the thrust velocity decreased from about 10 cm onwards. At a closer range (<5 cm), this was associated with a slight increase of the fEOD. This inverse relation between object-nearness and thrust velocity as well as the link between nearness and fEOD were also evident for trajectories extracted with inverting the spatial filters best described as “departure from object” (*n* = 389; Figure S6). To determine the behavior following an “object approach” chain, we calculated the transition probabilities based on the ten frames following an “object approach” chain (Figure S7). The transition probability to PM 01 was significantly increased, indicating that object exploration behaviors of low thrust, including “stationary behavior” or “backwards swimming,” are likely to occur after the approach sequences. Moreover the transition probabilities to PM 02 and PM 07/08 were significantly increased, which indicates that the SPM was probably followed by kinematics similar to those that contributed to SPM 08 itself (see also Figure 3C).

### Sequences of behavior

**Figure 6:**
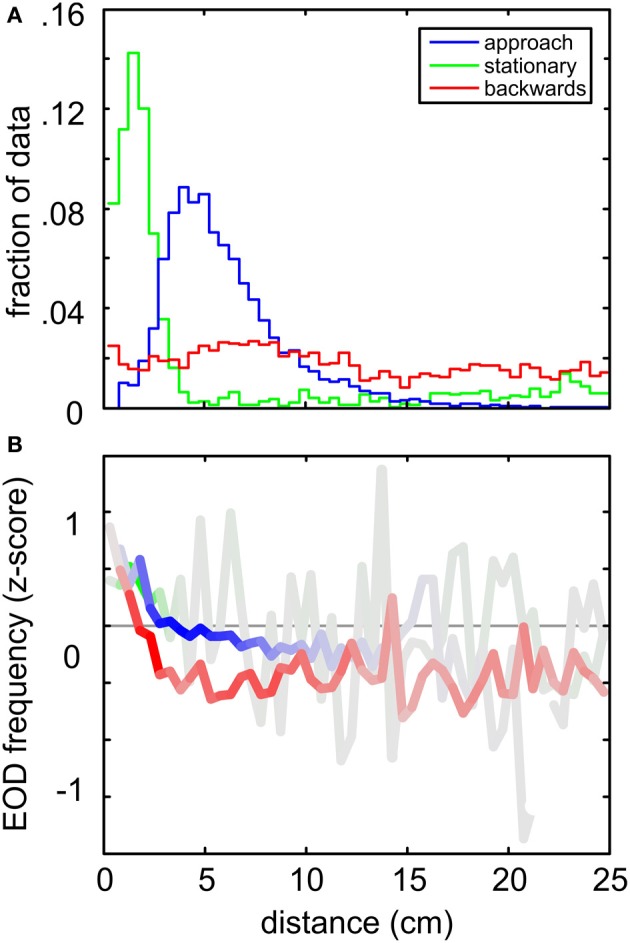
**Comparison between the spatial distributions and the EOD frequencies for the three behaviors analyzed in detail. (A)** Relative spatial distribution of “object approach” (blue), “stationary behavior” (green) “backwards swimming” (red) behaviors. Data was binned (bin width 1 cm) with respect to the Euclidian distance where the behavioral patterns were found. The frequency of occurrence is expressed relative to the size of the complete dataset (*n* = 277585 frames) at each distance. Stationary and object approach behaviors both peaked close to the object, while backwards swimming behavior was roughly equally distributed. Note that the classification of backwards and stationary behavioral patterns was based on kinematic data exclusively. **(B)** Averaged z-scored EOD frequencies occurring during the three behaviors as a function of distance to the cube. The color intensity indicates the relative amount of data contributing to the individual means. Note that for all behaviors an increase in fEOD toward the object is visible.

When comparing the three quantitatively characterized behaviors (“stationary behavior,” “backwards swimming” and the “object approach”) it is interesting to note that they occurred with differing spatial relation to the object. “Object approach” and “stationary behavior” both were object centered behaviors, whereas chains of “backwards swimming” showed a less strong increase of frequency of occurrence toward the object (Figure 6A). Note, however, that for the “object approach” behavior the spatial relation to the object was part of the applied filter, and thus it was not unexpected to find it being object-centered. The relation of the peak distances between “object approach” and “stationary behavior” indicated that they frequently occur in temporal succession, thereby forming a joint sequence of object approaching and inspection behavior (see Figure S3, and for an example of such a transition, see Figures 3A,C). This illustrates the potential of quantitatively extracting even more complex behavioral sequences on a higher level. All three behaviors showed an increase in the z-scored fEOD with nearness to the object (Figure 6B). Here the relative amount of data at each distance is coded in the intensity of the color (gray to full color), showing that the increase in the frequency in a range below 5 cm is fairly robust.

In summary we here have shown that different types of behavior can be objectively determined on differing time and complexity-scales (single frames to full behavioral sequences). Kinematic building blocks thus may be combined to analyze the superstructure of complex behavioral sequences. To illustrate this, using the quantitatively described behaviors introduced above, we show the temporal order of all the behaviors in an exemplary longer behavior of roughly 10 s duration in Figure 3 (“object approach”: blue; “stationary behavior”: green; “backwards swimming”: red; “object departure”: yellow). Note that these behaviors together form a longer behavioral sequence with only few kinematic transitions in between. For the major aim of capturing kinematic patterns of electrolocation behaviors in order to quantitatively analyze their sensory flow, such transitions can be neglected. Even for these short individual behaviors the fEOD showed the features described for the triggered averages of the kinematic chains above, i.e., during the “object approach” the frequency increased, during the “stationary behavior” probing was characterized by slight unsteady elevations of fEOD above the average, while during “backwards swimming” the fEOD was relatively low and constant (Figure 3B).

The shown example sequence indicates that the choice of the parameters to define chains of behavior requires some degree of deterministic user-interaction (here kinematic thresholding and spatial filtering of SPM08). To which degree the chosen parameters prove suitable will depend how variable or stringent an investigated behavior reoccurs within ones dataset. In addition, larger datasets and the creation of more super-prototypical chains should make the creation of seamless quantitative behavioral classification possible—however, this was not attempted in the present study.

Despite these limitations, the quantitative approach used here offers a lot of potential both to better quantify the kinematics in a specific context and, in addition, to now use these quantified kinematic sequences to explore and model the interaction between the movements and the electrosensory flow induced both by these movements and environmental factors.

## Discussion

Following a general definition of active sensing, which defines it as an expenditure of energy for the purpose of sensing (Nelson and MacIver, 2006), movements are crucial components of active sensing strategies. The hypothesis motivating our study is that such movements may be used to generate and shape the spatiotemporal sensory flow such that it matches the current task—either in terms of motor efficiency or regarding information gain. In that sense active sensing would involve all strategies which purposefully change the sensors' state according to the current sensing strategy (Bajcsy, 1988).

For electrolocation in Gymnotiform weakly electric fish, which emit a continuous quasi sinusoidal electric signal, MacIver and co-workers showed that specific movement strategies can increase the sensory volume available to the animals, thereby enhancing prey detectability (MacIver et al., 2001, 2010; Snyder et al., 2007). This is reminiscent of the way in which bats were shown to enlarge their acoustic sensory volume in echolocation (Yovel et al., 2011) or task-dependent whisking patterns in rodents (Carvell and Simons, 1995). For electrocommunication in an Apteronotoid fish it has been shown that motion patterns lead to spatiotemporal correlations in the sensory input that may be matched with specific sensory needs during electrocommunication and electrolocation (Fotowat et al., 2012, 2013). Beyond that a variety of studies interpreted stereotyped motions in weakly electric fish as an active strategy to create or shape sensory input (Heiligenberg, 1975; Toerring and Moller, 1984; Nelson and MacIver, 1999; MacIver et al., 2001; Cowan and Fortune, 2007; Stamper et al., 2012; Yu et al., 2012; Hofmann et al., 2013b). These studies led to the hypothesis that animals execute specific motor patterns in order to create and analyze signals of differing spatial and temporal structure. Indeed, the differently tuned topographical maps of weakly electric Gymnotiform fish (for a recent review, see: Krahe and Maler, 2014) are differentially used for electrocommunication and electrolocation, two behaviors where the relevant electric signals differ in their spatiotemporal structure (Cowan and Fortune, 2007).

Although electric images can be ambiguous (Engelmann et al., 2008), conditioning experiments in *Gnathonemus* have shown that these ambiguities can be overcome (Fechler et al., 2012). This indicates that the animals make use of a spatiotemporal sequence of electric images, i.e., electric flow. Various cues that might allow information extraction from electric flow have been addressed in recent works (Nelson and MacIver, 1999; Chen et al., 2005; Babineau et al., 2007; Sim and Kim, 2011, 2012; Fechler and von der Emde, 2012; Hofmann et al., 2013a). As pointed out above, specific motor patterns are postulated to be linked with specific sensory-motor tasks. Using weakly electric Mormyrid fish has the benefit that their discretized sensing strategy allows to link the sensory signal with the occurring body motions directly. Repetitive motor patterns were qualitatively described and linked to the electrosensory probing of objects (Toerring and Belbenoit, 1979; Toerring and Moller, 1984; Von der Emde, 1992). The data presented here combines aspects of these classic qualitative studies from the field of electrolocation with quantitative methods for behavioral descriptions (Braun et al., 2010; Geurten et al., 2010). As a first step, we here present detailed descriptions of electrosensory behavior on a quantitative level. The established methods enable us to describe the behavior on different levels, from the level of pooled and single frame data without temporal context, to the level of single frame kinematics that lead to short behaviors and longer connected behavioral sequences under consideration of the temporal structure.

During object presentation the animals responded to the novel object by an altered spatial usage of the arena as well as with distance dependent changes in their fEOD. This is reflected in the kinematic quantification of the behavior on the single frame level, where different prototypes of motion were found to differ with respect to fEOD and the likelihood of occurrence with respect to the distance to the object.

In order to characterize longer behavioral sequences we analyzed repetitively occurring kinematic patterns of homogenous chains of PMs. Here short and sharp turnings of the animal as well as gliding patterns (Figures S4, S5) without a distinct spatial relation to the object were found for PM 02. This behavior is interpreted to constitute part of the general locomotor repertoire (Toerring and Belbenoit, 1979). A common active sensing strategy in vision is the separation of translational and rotational optic flow through extremely fast and short saccadic motor patterns (Collett and Land, 1975; Srinivasan et al., 1991; Voss and Zeil, 1998; Egelhaaf et al., 2012). We thus compared homogeneous PM chains of translational and rotational PMs (Figure S4A). Chains dominated by translational movements were in general longer and had a higher duration compared to those that were dominated by rotational PMs. Although relatively short, these turning behaviors were much slower and of low amplitude (~ 200–250°/s). Given that fish in general are capable of very fast turns (C-start up to 2000°/s, Eaton et al., 1977), the lack of chains which such high amplitudes may indicate that a separation of rotation and translation is not sought after in electrolocation. Rather the turns might be used by the fish to actively shift the electrosensory focus to different parts of the sensory mosaic, thereby maximizing the sensory-related input. However, this hypothesis requires a more detailed analysis, including modeling of the associated electric images.

Some of the probing motor acts described by Toerring and Belbenoit (1979) were only observed during active electrolocation, including the so called “(lateral) va-et-vient” behavior. During this PMA the animals execute translational back and forth movement alongside an object. This behavior might be sought to generate temporal slopes e.g., to extract the relative distance of an object (Babineau et al., 2007; Sim and Kim, 2012; Hofmann et al., 2013a). We extracted sequences of “backwards swimming” that resemble the main component of such “va-et-vient” PMAs (Figure 4D): a low but positive thrust velocity followed by a rapid deceleration in which thrust velocity reverses and stays in the negative range for about 750 ms before the animals retuned to low positive thrust values. Similar to the EOD emission pattern reported for va-et-vient (Toerring and Belbenoit, 1979; Toerring and Moller, 1984; Von der Emde, 1992), the inter-pulse-interval distribution during “backwards swimming” was increased and for the example sequence some regularization of fEOD is visible (see red dots in Figure 3B). Variations in the inter-pulse-interval directly impact the latency-code of the electroreceptor afferents (Sawtell et al., 2006). A regularized interval distribution might be beneficial to minimize this effect and thereby maintaining constant sensitivity.

The “stationary behavior” characterized here quantitatively resembles the PMA descriptions of “chin probing” and “stationary probing” (Toerring and Belbenoit, 1979). During this behavior the fish remained nearly motionless for about 500 ms. This was accompanied by an increase of the fEOD. Thus, while the fish altered their position only marginally in this phase (see also green dots in Figure 3B), the temporal dynamics of the sensory flow are variable. As this stationary behavior was frequently exerted close to the object even small movements of the body can lead to considerable alterations of the sensory input, as the amplitude of the obtained electrical images critically depends on distance due to the spherical spread of the carrier (Nelson and MacIver, 2006). Close to the object a considerable amount of direct haptic contacts between the movable chin appendix of *Gnathonemus* and the object can be expected. While our current study could not resolve these contact points, future studies should take those into consideration. Probably the near-field active electrosensory system and the haptic capability of the chin appendix are recruited together and contacts may be directed toward particularly salient properties of an explored object. Thus they may aid in further elucidating which electrosensory cues lead to haptic contacts.

Behavioral sequences of course can be more complex than the chains of homogeneous prototypes. We chose to characterize heterogonous kinematic patterns based on the transition probabilities between PMs. For this we reduced the complexity of the dominating transition patterns and obtained 17 “super-prototypical movements” (SPMs). Interestingly, besides differences in SPM length and their relative frequency of occurrence (Figure S3), the SPMs relative frequency of occurrence for nights with and without an object present differed (Figure 5A). This indicates that the kinematics employed by the animals depends on the environmental context and that SPM 08 is a behavioral pattern preferentially shown in presence of the object. By applying an additional spatial conditional to this SPM, we extracted “object approach” behaviors. Compared to the PMAs (Toerring and Belbenoit, 1979), this is comparable to aspects of the “tangential” and “chin probing” PMAs. Rather than being more diverse it seems that in presence of novel objects in the environment the animals showed less variable SPMs. One possible explanation of this effect could be that the objects provide sensory cues that trigger directed and stereotyped motor patterns.

The sensory input during such an approach is comparable to a looming stimulus, where the peak amplitude of the sensory input and the width of the electric signature increases in a power law fashion (Sanguinetti-Scheck et al., 2012; Clarke et al., 2013). The strongest increase in the electrosensory input can be expected at low object distances. At these short distances fish showed an increase in the fEOD. Such an increase may reflect a compensatory mechanism by which the relative change in the sensory input of successive electric images is reduced, leading to a stabilization of the rate of change in the sensory input. This relation between fEOD and distance was found for trajectories in which the fish departed an object (Figure S6) as well as for the other object-centered behaviors (Figure 6). “Object approach” trajectories were found to be frequently followed by PMs that have been shown to play a dominant role during sensory relevant behaviors (Figure S7). This and the example sequence shown in Figure 3.emphasize that stereotyped behaviors can be sequentially arranged to represent and categorize more complex “behavioral sequences.” While the underlying kinematics probably are fairly constant, this shows that fish may actively alter the composition of such longer sequences to match their current motor and sensory requirement. In summary, comparing the published qualitative PMAs with our quantified behaviors we show that it now is possible to quantitatively extract complex behavioral patterns in electrolocation.

A key parameter for electrolocation in pulsatile species is to understand how the inter-pulse-interval contributes to the sensory flow. Our results show that this relation may differ depending on the context. Notably this was found to be the case already at the single-frame level of PMs. The correlation between fEOD and kinematic velocities implies that some direct motor-sensory interaction exists that links the motor system with the electromotor component of active electrolocation. This is in accordance with a recently established direct motor-control network through which the Mauthner cell system can adjust the fEOD (Comas and Borde, 2010). The fEOD was further found to increases during “object approach” sequences, where the kinematic values decreased with nearness to the object while the strength of the sensory input increases dramatically. This is reminiscent of other active sensory systems like the vibrissal system (Friedman et al., 2006), echolocation in bats (Ulanovsky and Moss, 2008; Yovel et al., 2011) or active olfaction (Schroeder et al., 2010). One possible interpretation of this increase may be that it could partially compensate for the step change in the sensory input increasing heavily during “object approach.” Contrary to the mechanism for motor control of EOD emission rates considered above, the underlying control mechanism in this case can be assumed to be based on the sensory input.

To unravel the consequence of both the motor and sensing strategies will require analyzing the sensory flow associated with these strategies in depth. Therefore one of our main aims will be the modeling of the electrosensory input during the now kinematically quantified naturalistic behaviors. Additionally it will be of interest to specifically follow up on the idea that fish seek active motor strategies in an optimal fashion. Therefore behavioral experiments using the well-established learning paradigm (Von der Emde et al., 2010) could be combined with our kinematic analysis to investigate if and how the kinematics and active sensing strategies change during electrosensory-based learning.

## Author contributions

Jacob Engelmann and Volker Hofmann designed the study; Volker Hofmann conducted the experiments; Volker Hofmann, Bart R. H. Geurten, Jacob Engelmann, Juan I. Sanguinetti-Scheck, and Leonel Gómez-Sena analyzed the data; Volker Hofmann and Jacob Engelmann wrote the manuscript with intellectual contributions from all other authors.

### Conflict of interest statement

The authors declare that the research was conducted in the absence of any commercial or financial relationships that could be construed as a potential conflict of interest.
