# Supplementary material for: Motor patterns during active electrosensory acquisition
Source: Front Behav Neurosci. 2014 May 28;8:186. doi: 10.3389/fnbeh.2014.00186 (PMC4036139; doi:10.3389/fnbeh.2014.00186)
Supplement: Supplementary file 1 [file Presentation1.ZIP › Presentation /92855_Hofmann_Manuscript_Supplementary Material.docx]

**Supplementary Material**

**Supplementary Figure 1 Experimental setup.**The experimental setup was an 80 cm x 80 cm Perspex tank filled with 5 cm of water. Two plastic tubes (r = 3 cm) were placed as shelters parallel to the sidewalls. The tank was illuminated from below (880 nm LEDs) using a frosted glass to diffuse the light. The camera view covered an area of 64 cm x 48 cm and was centered on the middle of the tank. To record EOD signals, an array of eight silver wire electrodes was attached to the tank walls, with the electrode tips 2 cm above the tanks floor (“electrode arrangement”). Signal geometry of the two primary differential amplifier stages were arranged perpendicular to each other (see “recording scheme”) to prevent EODs from being missed by triggering due to movements of the fish. EOD events triggered the frame grabbing by a fire wire camera positioned on top of the tank. Recording procedures were carried out with custom written MATLAB routines.

**Supplementary Figure 2 Determination of parameter space for the clustering.
(A)** After determining the potential range of cluster classes by an agglomerative hierarchical clustering (see methods) we evaluated the stability of the results of a k-means clustering for k = 2 to k = 50. During k-means classification the initial value for a centroid is randomly determined and sequentially optimized. We clustered the data 100 times and evaluated the distance of the cluster centroids obtained each time to determine the stability of the classification. The reproducibility of the clustering was also tested through using only parts of the dataset (90, 75 & 50% see legend in B). For k = 10 we obtained a local minimum in the instability of both the reduced and full dataset. **(B)**The quality of the classification was evaluated as the ratio of the outer and the inner cluster distances. The outer centroid distance was determined as the minimum Euclidian distance between the given and all other centroids. The inner distance was calculated as the mean distance between a centroid and all values scored to belong to this cluster. For k = 10 we found the quality to be ≈ 3.2 which indicates that the mean distance between centroids is 3.2 fold compared to the mean inner distance of the centroids.

**Supplementary Figure 3 Characterization of the 17 super-prototypical movements obtained from the dataset.
(A)** Schematic showing the PM composition of the different SPMs. For illustrative purposes we here reduced constant PM segments to a single circle, thereby the chain of circles illustrates the consecutive chain of PMs constituting the individual SPMs. Only one possible kinematic combination of each SPM is shown. **(B)** Mean durations of the SPM chains (bars = mean; error bars = ± one standard deviation). **(C)** Absolute number of sequences found for each SPM. In total the 17 SPMs cover 43.5% of the total frames in our dataset.

**Supplementary Figure 4 Spatial and kinematic characterization of sharp turns during swimming.
(A)** Comparison of the chain length and duration of PM chains that were mainly translational (PM 03/04 and PM 05/06) to those dominated by rotational components (PM 07/08 and PM 09/10). Translational PM chains on average were longer in chain length and duration (Wilcoxon/ Mann-Whitney U-test, p = 0, z = 8/29.3). **(B)** Sharp turns were evenly distributed across the arena with a halo of decreased probability around the object. Data is plotted as a two dimensional histogram with a bin width of 1 cm^2^. **(C)** Averages of kinematic and electromotor parameters (from top to bottom: thrust, slip and yaw velocity and the z-scored fEOD; see also Fig. 4 for detailed descriptions) triggered by the start of the target PM chain (2324 chains, n = 10015). Slip and yaw velocity showed clear deviations from the preceding framesat the time of the PM-chain start while the thrust velocity transiently and weakly increased. The fEOD increased weakly in conjunction with the rotational movements. **(D)** Same as C. but for right turns (2071 chains, n = 9027).

**Supplementary Figure 5 Short homogenous chains of PM 02 mark transitions between PMs, while longer homogenous chains of PM 02 indicate a “gliding” pattern.
(A)** Transition probabilities between PMs prior to and after homogenous sequences of PM 02 for short chains (upper panel) and long (>10 frames) PM 02 chains. For longer chains the transition were significantly increased from and to PM 02 and PM 07/08. For shorter chains the transition probabilities to PM 05/06 were significantly increased as well, indicating that some of these short PM 02 chains are involved in chains of translational swimming, i.e., alterations of rotational and translational PMs. **(B)** Averaged kinematic and electromotor parameters triggered on the beginning of the long homogenous PM 02 chain (829 chains, n = 12989). As expected for a gliding behavior, the variability of all kinematic parameters decreased. While the means of slip and yaw velocities were constant around zero, the mean thrust velocity decreased transiently. The fEOD showed only a short and weak increase around the start of the PM chain.

**Supplementary Figure 6 Characterization of kinematic and electromotor patterns classified as “object departure”.
(A)** Lollipop plot of 50 randomly chosen “object approach” chains (same plot as Fig. 5e). **(B)** Same as in (A), but for sequences of “object departure”. To detect these sequences the conditionals used to determine “object approach” sequences were inverted (distance needed to increase for sequences starting close to the object). This resulted in trajectories that were similar to the “object approach” sequences, but were of opposite heading of the animal. **(C)** Averages of the kinematic and electromotor parameters for “object departure” chains (383 chains, n = 7239). Note that here the average was calculated with the object distance as a frame of reference. The thrust-velocity shows an increase with increasing object distance, while slip- and yaw-velocity did not show a clear relation to the distance to the cube. The fEOD decreases with increasing object distance. Note that these characteristics were completely parallel to those of “object approach” sequences.

**Supplementary Figure 7 PM-transition probabilities following the “object approach” sequences.**The transition probability for a given PM was calculated from the ratio of the actual occurrence of this PM in the 10 frames after the approach SPM. The expected probability of the PMs is based on its frequency of occurrence in the whole dataset. PM 02, PM 07/08 and PM 01 occurred more frequently than expected, while all other PMs were less frequently observed after the approach sequence than expected. This indicates that following the approach pattern, fish were more likely to show exploratory behaviors like “stationary behavior” and “backward swimming” (PM 01).
