# Supplementary figures and images for: Motor patterns during active electrosensory acquisition
Source: Front Behav Neurosci. 2014 May 28;8:186. doi: 10.3389/fnbeh.2014.00186 (PMC4036139; doi:10.3389/fnbeh.2014.00186)

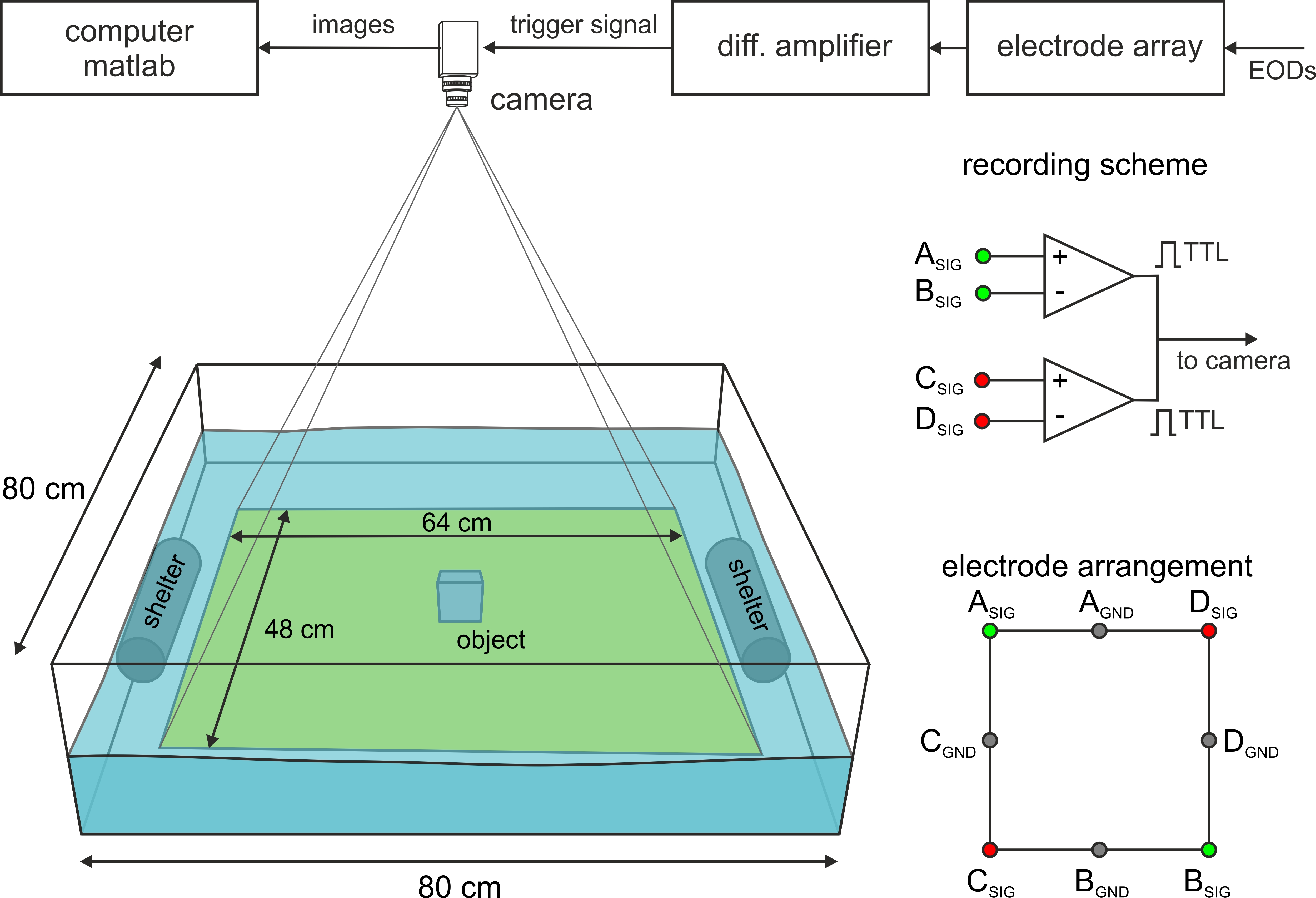

Supplement: Supplementary file 1 [file Presentation1.ZIP › Presentation /92855_Hofmann_Figure_1.JPEG]

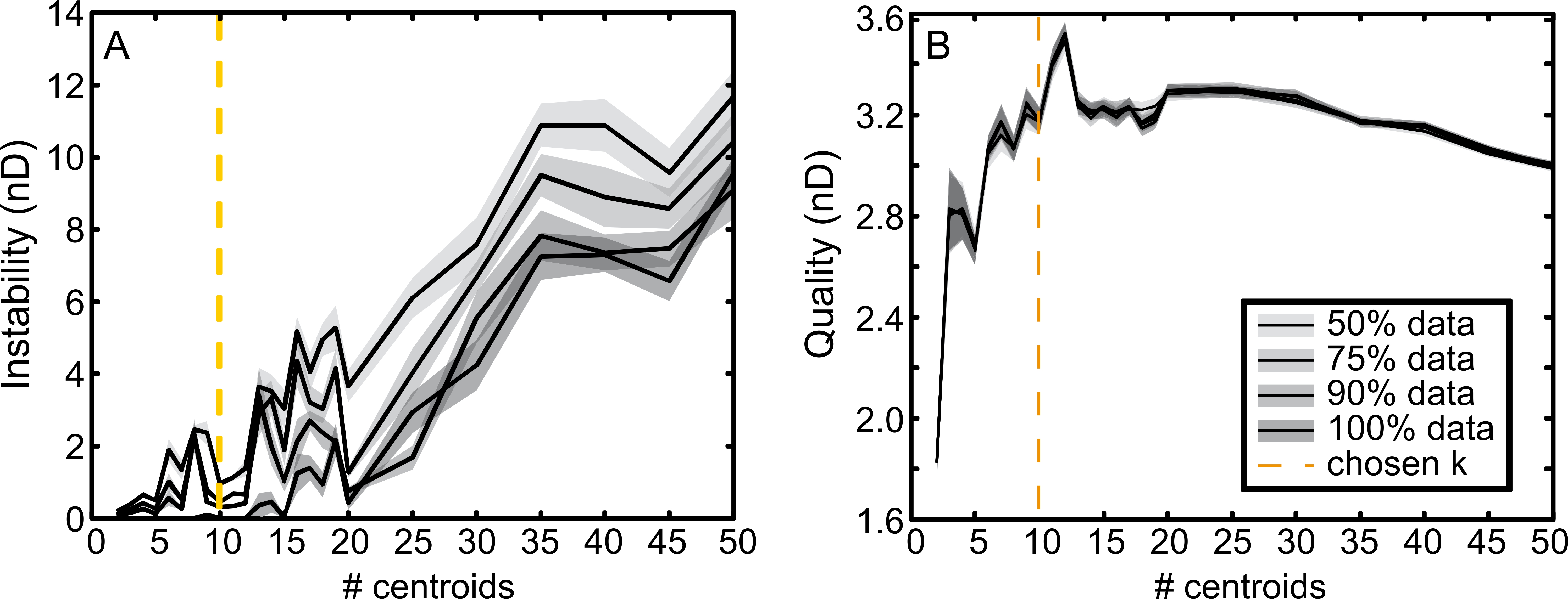

Supplement: Supplementary file 1 [file Presentation1.ZIP › Presentation /92855_Hofmann_Figure_2.JPEG]

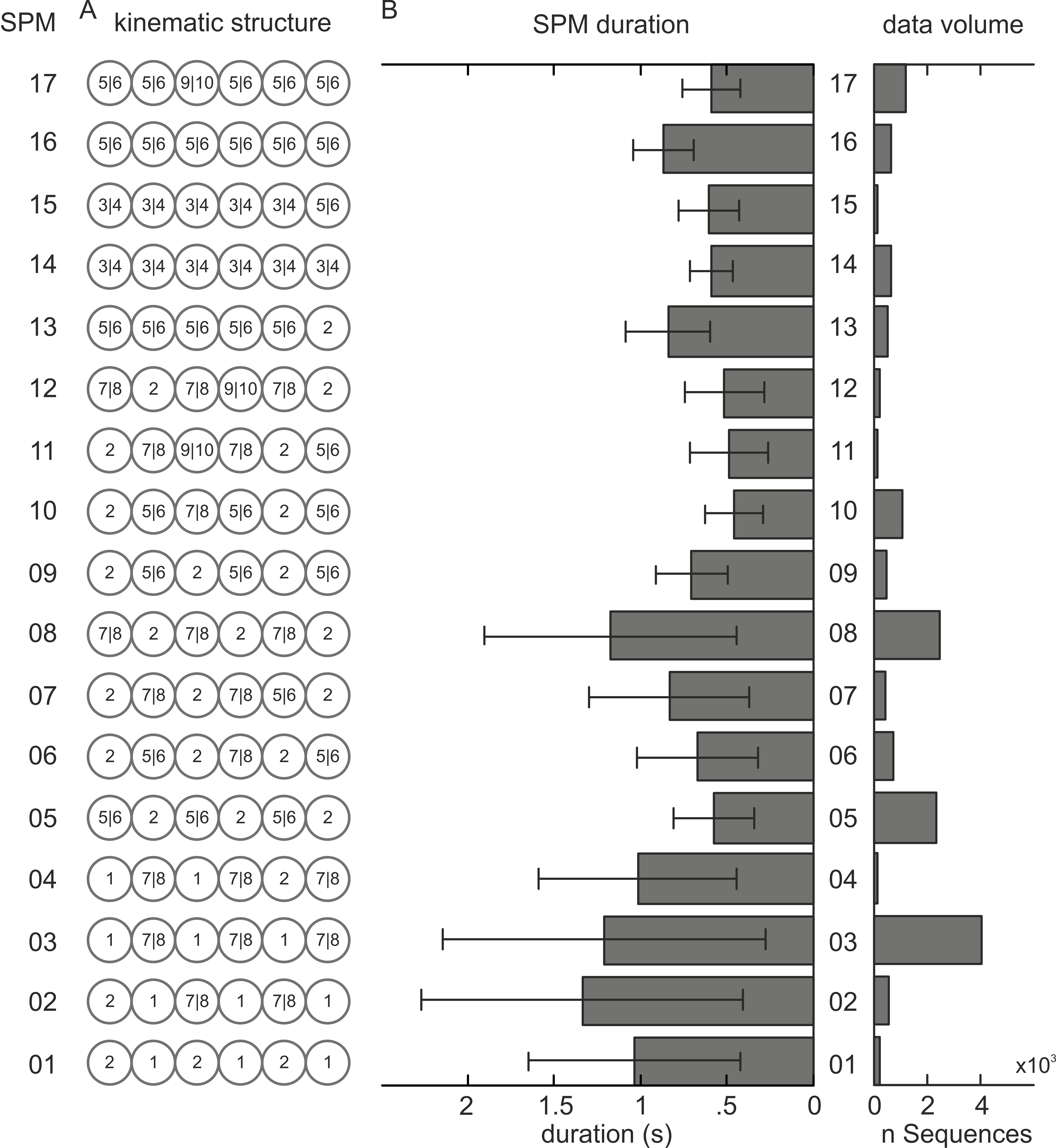

Supplement: Supplementary file 1 [file Presentation1.ZIP › Presentation /92855_Hofmann_Figure_3.JPEG]

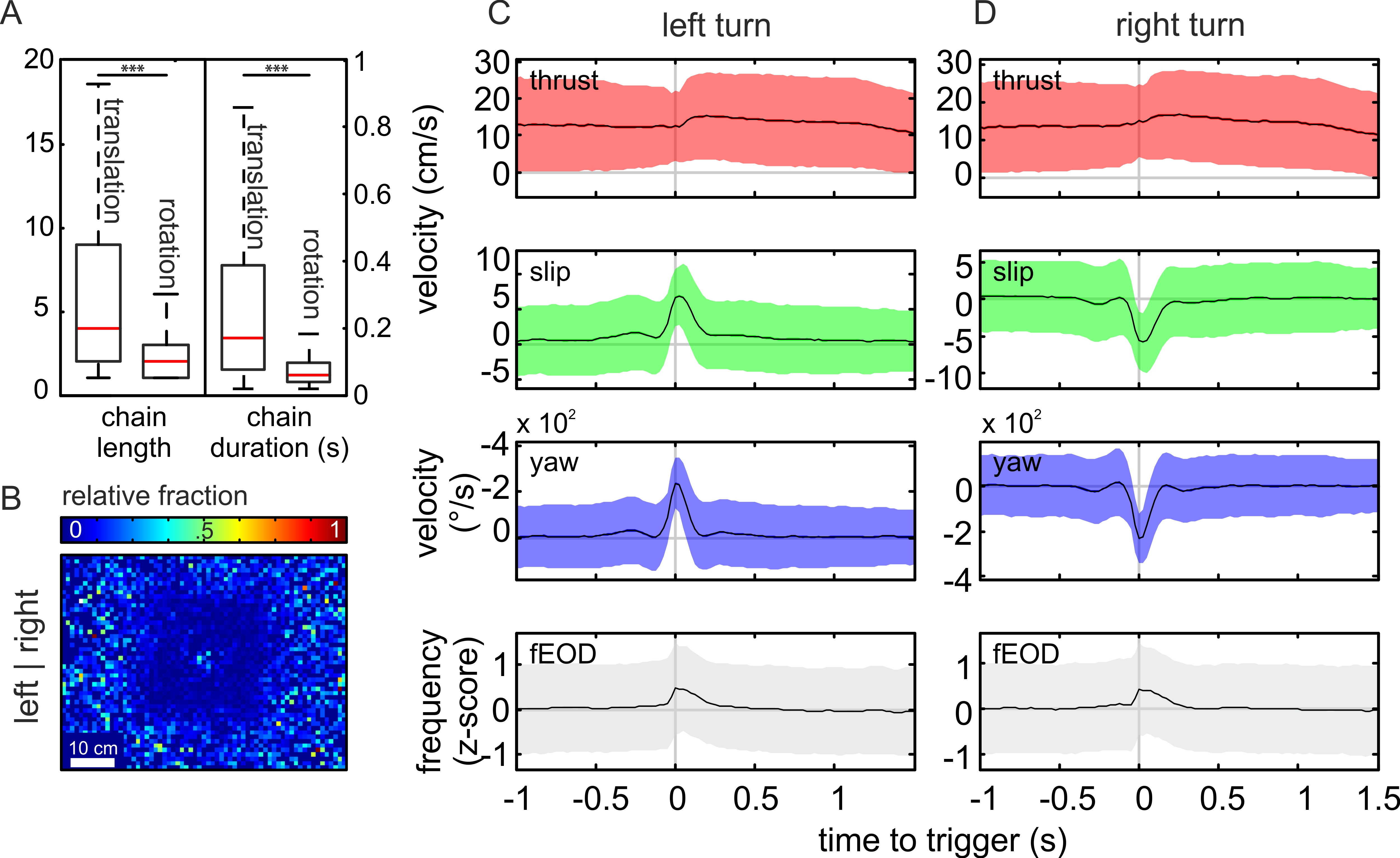

Supplement: Supplementary file 1 [file Presentation1.ZIP › Presentation /92855_Hofmann_Figure_4.JPEG]

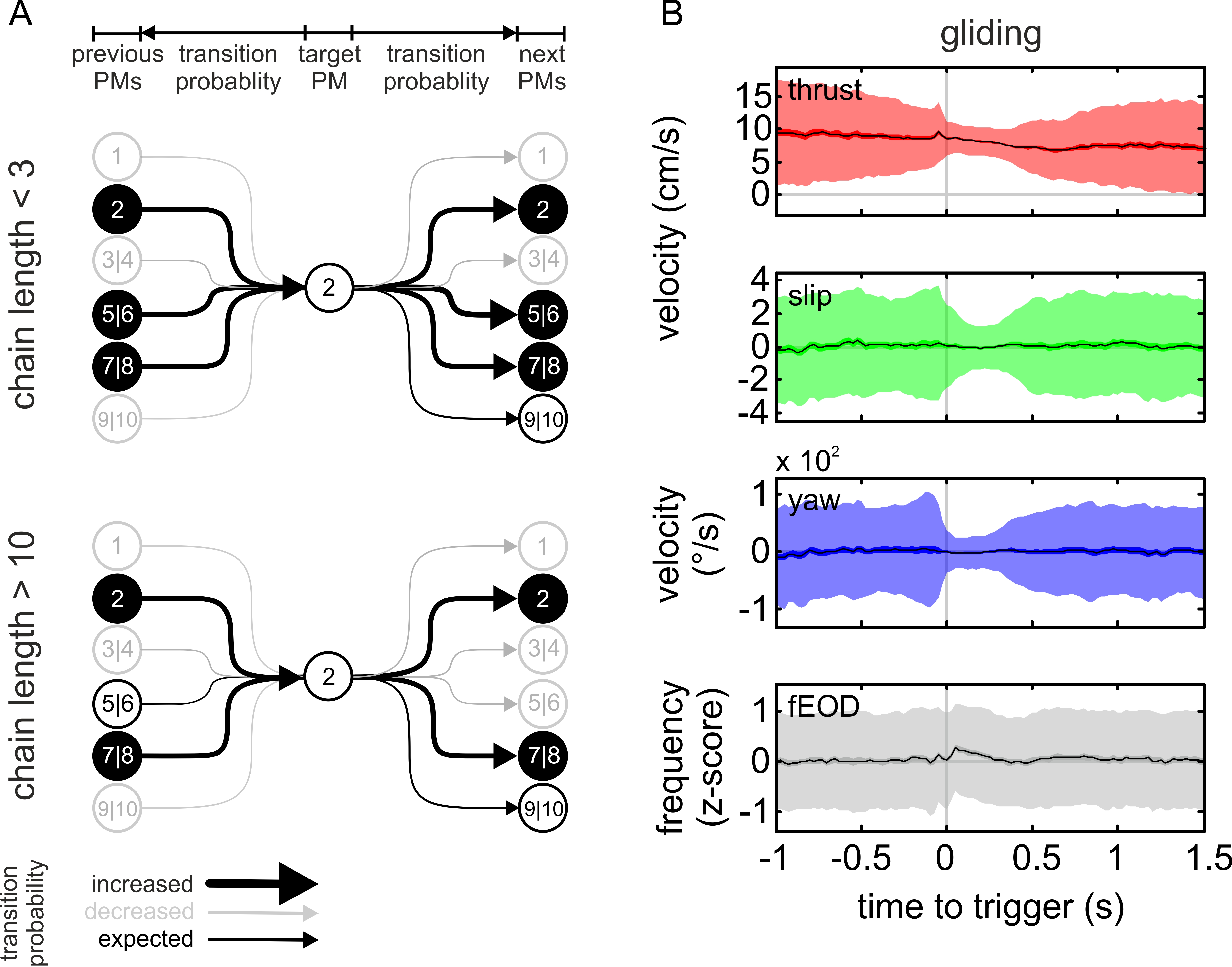

Supplement: Supplementary file 1 [file Presentation1.ZIP › Presentation /92855_Hofmann_Figure_5.JPEG]

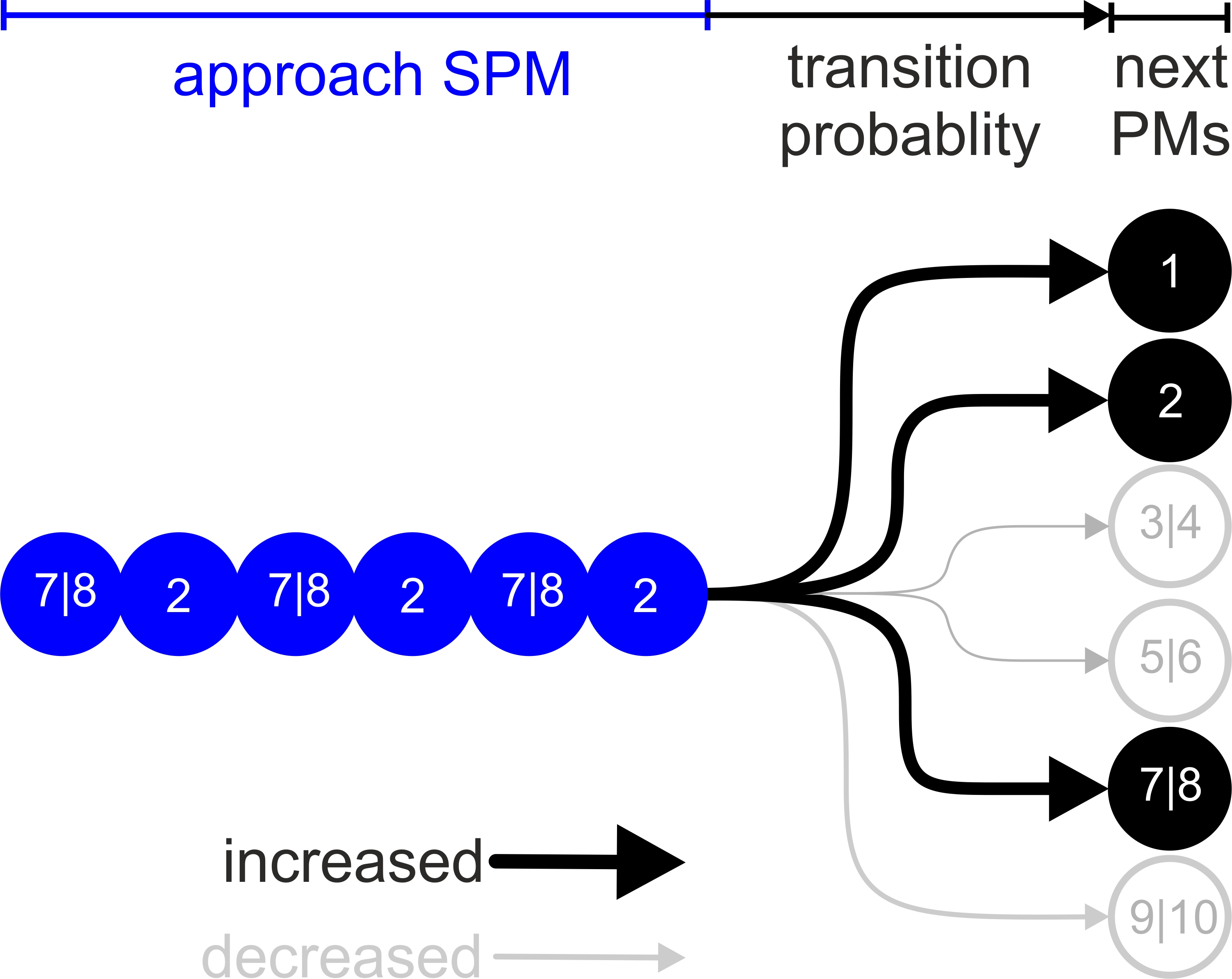

Supplement: Supplementary file 1 [file Presentation1.ZIP › Presentation /92855_Hofmann_Figure_6.JPEG]

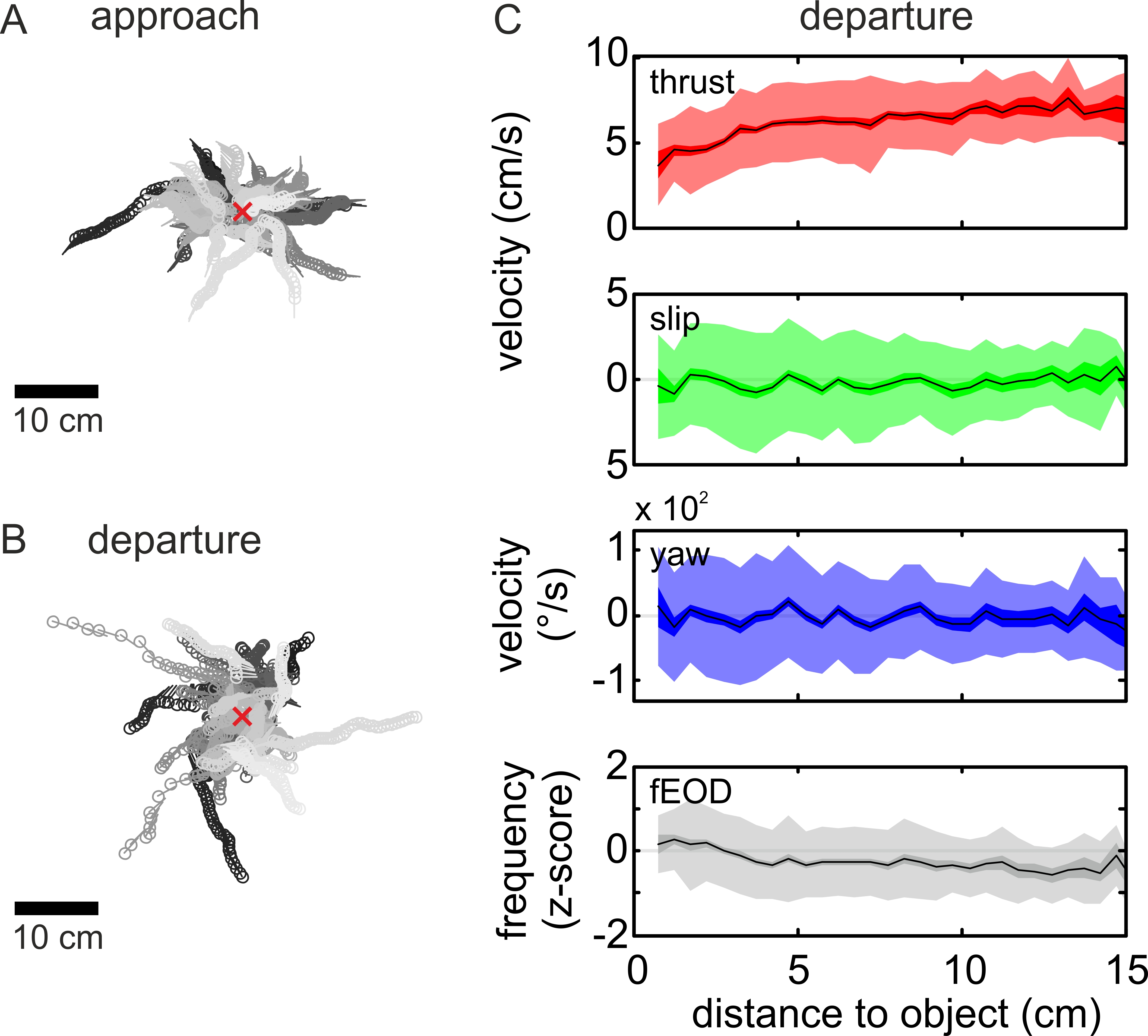

Supplement: Supplementary file 1 [file Presentation1.ZIP › Presentation /92855_Hofmann_Figure_7.JPEG]
